# Columnar Mesomorphism in a Methylthio-Decorated Triindole for Enhanced Charge Transport

**DOI:** 10.1021/acsaelm.4c00693

**Published:** 2024-06-11

**Authors:** Constanza Ruiz, Raúl Martín, Angela Benito, Enrique Gutierrez, M. Ángeles Monge, Antonio Facchetti, Roberto Termine, Attilio Golemme, Berta Gómez-Lor

**Affiliations:** † Instituto de Ciencia de Materiales de Madrid, CSIC, Cantoblanco 28049, Madrid, Spain; ‡ School of Materials Science and Engineering, Georgia Institute of Technology, Atlanta, Georgia 30332, United States; § Faculty of Chemical and Technologies Sciences, University of Castilla-La Mancha, 13071 Ciudad Real, Spain; ∥ CNR Nanotec UOS Rende, Dipartimento di Fisica, Università della Calabria, Rende 87036, Italy

**Keywords:** Organic semiconductors, discotic liquid crystals, OFETS, SCLC measurements, hole mobility

## Abstract

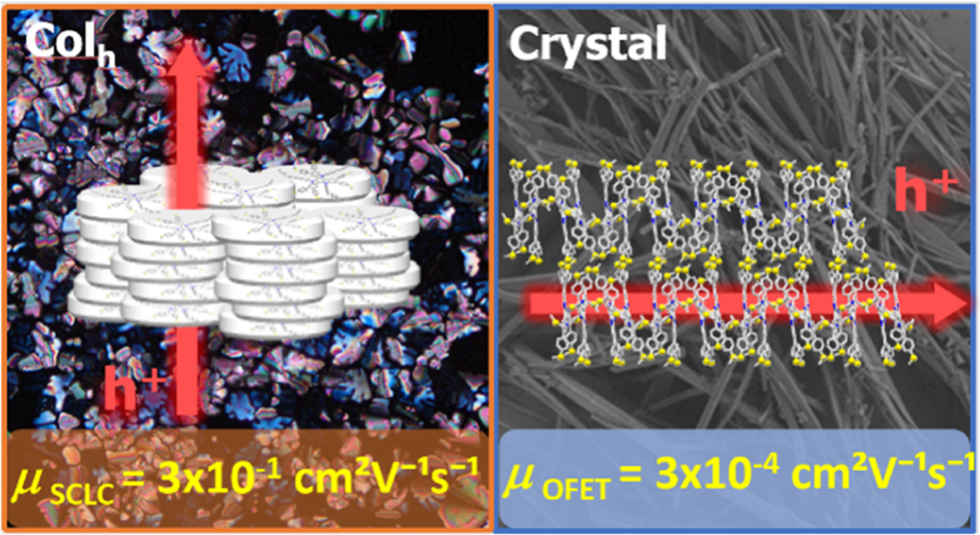

We report a semiconducting triindole-based discotic liquid crystal (**TRISMe**) functionalized with six *p*-methylthiophenyl groups at its periphery. While initially a crystalline solid at room temperature, **TRISMe** transitions to a columnar hexagonal mesophase upon heating and retains this supramolecular organization upon subsequent cooling, despite having only three flexible alkyl chains attached to the core’s nitrogens. The incorporation of methylthio groups effectively hinders tight molecular packing, stabilizing the columnar arrangement of this disk-shaped molecule. Single crystal analysis confirmed the high tendency of this compound to organize into a columnar architecture and the role played by the methylthio groups in reinforcing such structure. The mesomorphic behavior of **TRISMe** provides an opportunity for processing from its molten state. Notably, our research reveals significant differences in charge transport depending on the processing method, whether solution drop-casting or melt-based. **TRISMe** shows hole mobility values averaging 3 × 10^–1^ cm^2^ V^–1^ s^–1^ when incorporated in diode-type devices from the isotropic melt and annealed at the mesophase temperature, estimated by SCLC (space-charge-limited current) measurements. However, when integrated into solution-processed organic field-effect transistors (OFETs), crystalline **TRISMe** exhibits a hole mobility of 3 × 10^–4^ cm^2^ V^–1^ s^–1^. The observed differences can be attributed to a beneficial supramolecular assembly achieved in the mesophase in spite of its lower order. These results emphasize the material’s potential for applications in easy-to-process electronic devices and highlight the potential of methylthio moieties in promoting columnar mesophases.

## Introduction

Attractive noncovalent interactions involving sulfur atoms have been marginally investigated and only now their potential in the stabilization of both biological and synthetic systems have been fully appreciated.^1,2^ Interactions such as S···S, S···O, S···N or S···π, CH···S, among others have demonstrated to play crucial roles for example in ligand-protein interactions^3,4^ or the conformation^5,6^ of polymers and organic molecules with fundamental implications in fields as varied as drug-discovery,^7^ molecular recognition,^8^ catalysis^5^ or materials science.^9−13^ Recently, the generality of nontraditional C–H···S hydrogen bonding as a powerful driving force in the self-assembly of organic materials has been highlighted.^1,14^ The propensity for S acceptors to make longer contacts with C–H donors, compared to those typically observed for N, O, and F, has been suggested as the probable reason for the historically overlooked importance of this interaction.^1^ The remarkable stabilities of C–H···S hydrogen bonds have mainly been attributed to attractive dispersion interactions.^14^

Sulfur containing structures are also gaining increasing interest in the molecular design of materials for organic electronics.^1,10,11,15^ In particular, the attachment of alkyl chains through sulfur linking atoms has been found to be an useful strategy for controlling the short-range order, packing arrangement and orientation of molecules in organic semiconductors, to improve their charge carrier mobilities.^11,15^

In the quest for high mobility molecular systems, discotic liquid crystals has been long envisaged as promising candidates^16−19^ In columnar mesophases, conjugated molecules are arranged in stacks that provide a suitable pathway for charge migration.^20−23^ Despite the remarkable charge transport properties of semiconducting discotic liquid crystals (mobility values above 8.8 cm^2^ V^–1^ s^–1^ and 6 cm^2^ V^–1^ s^–1^ have been reported for holes and electrons, respectively)^24,25^ their use is not as widespread as might be expected. The reason lies in their characteristic structure, which is built from molecules surrounded by long flexible alkyl chains.^26,27^ This structure induces phase segregation, promoting the arrangement of aromatic cores in columns favorable for efficient intracolumnar transport, but also results in melted alkyl chains forming an isolating cover, preventing intercolumnar charge transport. Consequently, the charge transport is highly uniaxial, making it highly dependent on the alignment of the columns on the substrates.^28−30^ While obtaining the necessary macroscopic orientation of columns on different substrates has been successfully achieved through diverse strategies, including the application of electric fields,^31^ shearing,^32^ surface treatments,^33^ or confinement effects,^34^ solutions to date vary across different materials and it still poses a notable challenge for the fabrication of easily processed devices. To reduce the alignment dependence, discotic molecules with less alkyl chains are preferred,^30,35^ thereby leading to discotic mesophases with a high conductor-to-insulator ratio.

The heptacyclic 10,15-dihydro-5*H*-diindolo[3,2-*a*:3′,2′-*c*]carbazole (triindole) disk-like semiconducting scaffold has been extensively investigated in the search of high performance *p*-type semiconducting discotic liquid crystals.^30,36−39^ Direct attachment of six peripheral decyl chains to this electron rich platform successfully induces the formation of highly fluid but poorly ordered columnar hexagonal mesophases.^39^ On the contrary, distancing the peripheral alkyl chains by phenyl linkers moieties can efficiently interlock the molecules within the columns yielding highly ordered mesophases.^36^ In this manuscript we study the effects of six peripheral *p*-methylthiophenyl groups on the mesomorphic behavior of *N*-dodecyl hexaaryltriindole (compound **TRISMe**). Interestingly, we have found that **TRISMe** endowed with only three long C12 alkyl chains attached to the nitrogen, forms a stable columnar hexagonal mesophase in a broad range of temperatures. Apparently, despite the reduced size of the methyl groups, the conformational and steric effects of the alkylthio groups are sufficient to hinder molecular packing while filling the free space around the molecules stabilizing the columnar arrangement, which makes it attractive in the search for discotic mesogens with low alignment dependence. The single crystal structure confirms the tendency of this compound to organize into columns and evidence the strong influence that the methylthio groups have in stabilizing such columnar arrangement. Finally, the semiconducting behavior of this compound has been demonstrated by estimating its hole mobility by means of two different methods: through space charge limited current measurements in a diode-type device with the semiconductor in a supercooled columnar mesophase by space-charge limited current measurements (μ_SCLC_ 3 × 10^–1^ cm^2^ V^–1^ s^–1^ ) or via field effect mobility measurements in crystalline films in a transistor device. (μ_OFET_ 3 × 10^–4^ cm^2^ V^–1^ s^–1^ ). Although these methods require a different conduction channel (perpendicular vs parallel to the substrate) and therefore have different alignment requirements (homeotropic vs planar), the large differences in mobility observed in this particular case are more likely due to the different phase in which the material is measured (mesophase vs crystalline) and the more favorable supramolecular arrangement of the discotic mesophase.

## Experimental Section

### Synthesis of 2,3,7,8,12,13-hexakis-(4-(methylthio)phenyl)-5,10,15-tridodecyl-10,15-dihydro-5*H*-diindolo[3,2-*a*:3′,2′-*c*]carbazole (TRISMe)

A mixture of *N*-dodecyl hexabromotriindole (100 mg, 0.07 mmol), (4-methylthio)phenyl boronic acid (91 mg, 0.54 mmol) and Pd(PPh_3_)_4_ (52 mg, 0.04 mmol) in 6 mL of THF and 1 mL of 2 M aqueous K_2_CO_3_ was carefully degassed and subsequently heated at 150 °C for 2 h in a microwave (MW) reactor. The mixture was cooled to room temperature, partitioned between H_2_O and CH_2_Cl_2_ and the organic phase dried over MgSO_4_. The solvent was evaporated and the compound was purified by column chromatography with CH_2_Cl_2_ as eluent to give **TRISMe** as a white solid (85 mg, 71%).^1^H NMR (300 MHz, C_2_D_2_Cl_4_, 25 °C, ppm) δ 8.21 (s, 3H), 7.54 (s, 3H), 7.21–7.19 (AA′BB′, 12H), 7.14–7.11 (AA′BB′, 12H), 4.82 (br t, 6H), 2.47 (s, 18H), 2.11 (m, 6H), 1.58 (m, 6H), 1.20–1.16 (m, 48H), 0.83–0.79 (br t, 9H). ^13^C NMR (75 MHz, CDCl_3_, 25 °C, ppm) δ 140.5 (C quart), 139.9 (C quart), 139.6 (C quart), 139.3 (C quart), 136.7 (C quart), 136.3 (C quart), 135.4 (C quart), 132.5 (C quart), 131.1 (2C, CH tert), 131.0 (2C, CH tert), 126.2 (4C, CH tert), 123.8 (CH tert), 122.7 (C quart), 111.8 (CH tert), 102.9 (C quart), 47.0 (CH_2_ sec), 32.2 (CH_2_ sec), 30.7 (CH_2_ sec), 30.0 (2C, CH_2_ sec), 30.0 (CH_2_ sec), 29.9 (CH_2_ sec), 29.8(CH_2_ sec), 29.7 (CH_2_ sec), 27.2 (CH_2_ sec), 23.0 (CH_2_ sec), 16.0 (CH_3_ prim), 14.5 (CH_3_ prim). UV–vis (CH_2_Cl_2_, 25 °C) λ_max_ 341 nm. MALDI-TOF MS *m*/*z* 1582.8 (M^+^); HRMS (MALDI-TOF) calcd for C_102_H_123_N_3_S_6_: 1582.8067 found: 1582.8054.

### Single Crystal Growth and Structure Determination of TRISMe

Crystals suitable for single-crystal analysis were obtained by slow diffusion of hexane in a THF solution of **TRISMe**. Crystals were selected under a polarizing optical microscope and glued on a glass fiber for a single-crystal X-ray diffraction experiment. Single-crystal X-ray data were obtained in a Bruker four circle kappa-diffractometer equipped with a Cu INCOATED microsource, operated at 45 W power (50 kV, 0.90 mA) to generate Cu Kα radiation (λ = 1.54178 Å), and a Bruker PHOTON 3 area detector. Single crystal X-ray diffraction data were collected exploring over a hemisphere of the reciprocal space in a combination of φ and ω scans to reach a resolution of 0.93 Å, using a Bruker APEX3 software suite. Unit cell dimensions were determined for least-squares fit of reflections with I > 20 σ. A semiempirical absorption and scale correction based on equivalent reflection was carried out. The structures were solved by direct methods. The final cycles of refinement were carried out by full-matrix least-squares analyses with anisotropic thermal parameters of all non-hydrogen atoms. The hydrogen atoms were fixed at their calculated positions using distances and angle constraints. A solvent mask was calculated and 496 electrons were found in a volume of 2768 Å in the void space. This is consistent with the presence of 62 CH_2_ per unit cell. Explanation to CheckCIF A alerts: Due to the low crystal diffraction at high angles, caused by the disordered position of the dodecane alkyl chains, some reflections with negative intensities were omitted. For this reason, the thermal parameter Ueq of C050 is higher than that of its neighbors and the distances H···H are not significant.

All calculations were performed using APEX3^40^ software for data collection and data reduction and SHELXTL^41^ and OLEX2^42^ to resolve and refine the structure, respectively. CCDC 2281315 contains the supplementary crystallographic data for **TRISMe**. These data can be obtained free of charge via www.ccdc.cam.ac.uk/data_request/cif, or by emailing data_request@ccdc.cam.ac.uk, or by contacting The Cambridge Crystallographic Data Centre, 12 Union Road, Cambridge CB2 1EZ, UK; fax: + 44 1223 336033.

### Diode Fabrication and SCLC Measurements

Cells were prepared by overlapping one glass with 3 Au stripes, on another one with 5 ITO stripes, obtaining 15 independent zones with an area of 0.6 mm^2^. The thickness of the resulting diodes was controlled by using 8 μm glass spacers and was always determined, before SCLC experiments, by interferometric measurements. The material was introduced by capillarity from its melt, slowly cooled to reach the mesophase and subsequently rapidly cooled to room temperature, in order to freeze the mesophase. The J/V curves were acquired by connecting the positive pole to the Au electrode and the negative pole to the ITO. A Keithley 6517A electrometer was used to obtain the Current/Voltage curves, while the capacitance of the cell was measured with an Agilent 4284A LCR meter.

### Field-Effect Transistor Fabrication and FET Measurements

To measure field effect charge carrier mobilities, transistors with a bottom gate and top contact configuration were fabricated. The gate/dielectric substrates (n-doped Si/300 nm SiO_2_) were cleaned in an ultrasonic bath with acetone, hexane and ethanol, dried under a flow of nitrogen and the surface and subsequently functionalized with a self-assembled monolayer of hexamethyldisilazane (HMDS). Semiconducting thin films were deposited by drop-casting a 40 μL of a 5 mg/mL solution of **TRISMe** in chloroform, under a nitrogen atmosphere. Finally, 30 nm gold source and drain electrodes were thermal evaporated through a shadow mask. Devices were tested under vacuum by using an Agilent B1500 semiconductor parameter analyzer and a customized vacuum probe station.

## Results and Discussion

### Synthesis and Structural Analysis of TRISMe

**Scheme 1:**
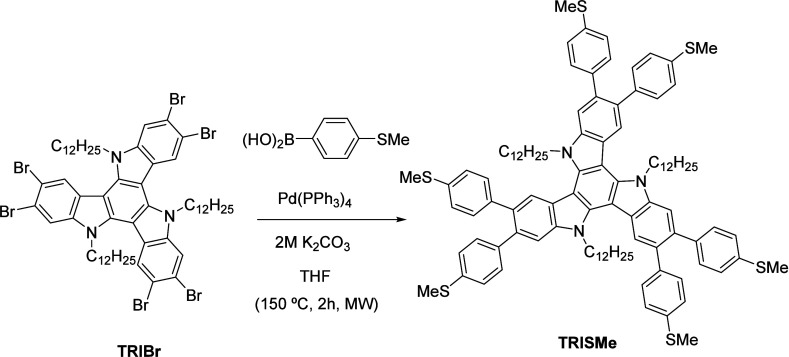
Synthesis of Compound **TRISMe**

Compound **TRISMe** was obtained by 6-fold Suzuki coupling of *N*-dodecyl hexabromotriindole **TRIBr** with *p*-methylthiophenyl boronic acid in the presence of Pd(PPh_3_)_4_ and K_2_CO_3_ 2M, using THF as solvent (Scheme 1). After column chromatogratographic purification in CH_2_Cl_2_ this compound was obtained as a white polycrystalline solid in a good yield (71%). Single crystals were obtained by slow diffusion of hexane in a THF solution of **TRISMe** and despite their small size, subjected to single crystal analysis. As expected for a mesogen, the dodecyl chains were largely disordered, and it was not possible to locate all 12 carbon atoms of the three chains from the difference Fourier maps. However, critical carbon atoms essential for the discussion, including those of the lateral *p*-methylthiophenyl groups, the central triindole moieties, and the alkyl chains proximal to the triindole core, were successfully located and anisotropically refined.

**Figure 1:**
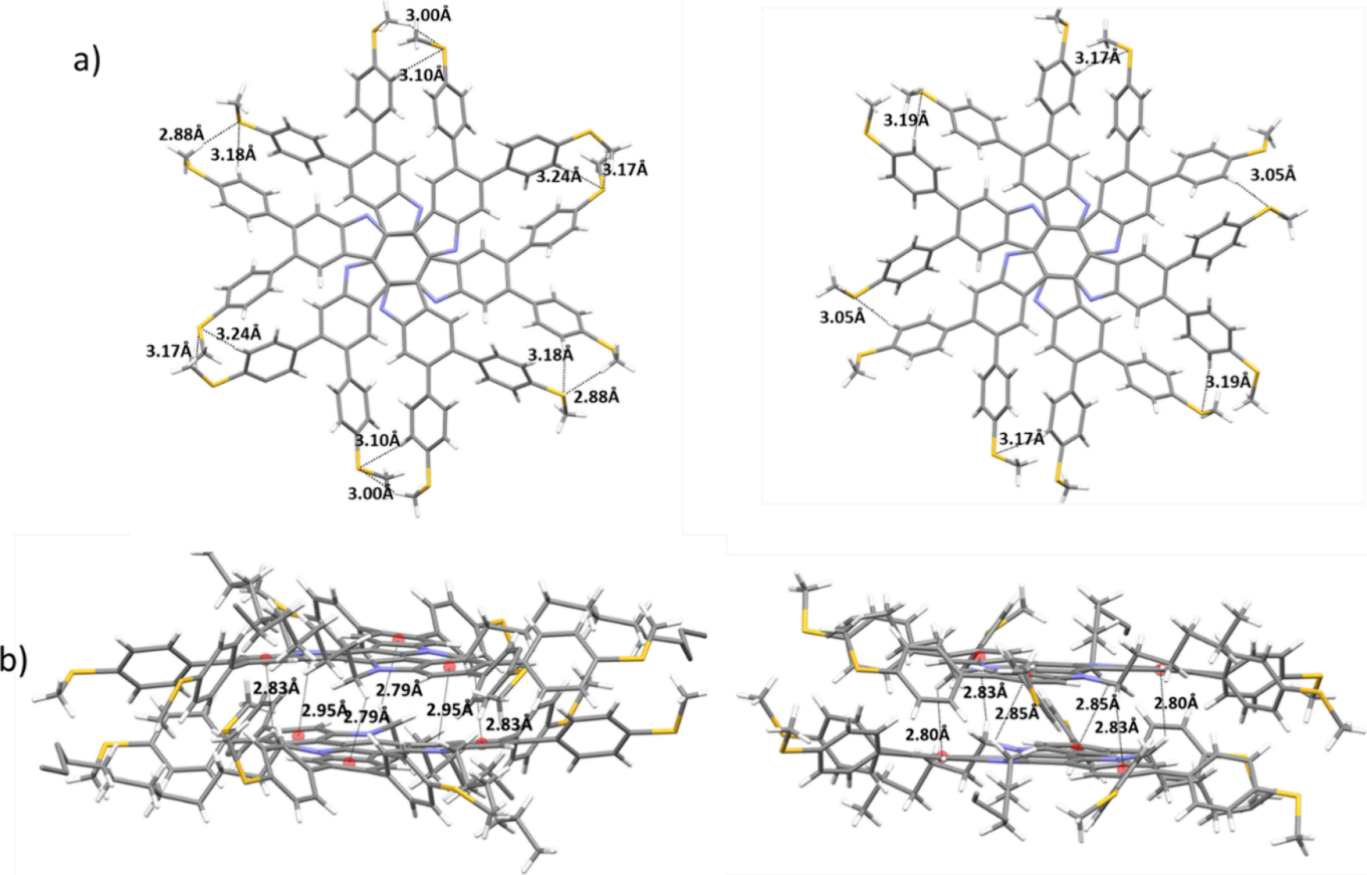
(a) Top views of the two different dimers alternating in the columns, and illustration of the short C–H···S contacts groups that stabilize them. The alkyl chains have been omitted for clarity. (b) Lateral view of the two different dimers alternating in the columns, illustrating the CH-π interactions, involving the strongly polarized CH_2_ groups attached to the nitrogen and the external phenyl rings.

The compound crystallizes in the triclinic P-1 space group, with lattice parameters of *a* = 19.54 Å, *b* = 23.23 Å and *c* = 24.31 Å and α = 67.72°, β = 81.92°, and γ = 73.74°. In the asymmetric unit there are two independent molecules which mainly differ in the geometry of the methylthio groups, that point toward distinct positions highlighting the flexibility of this moiety. Within the structure each molecule of the asymmetric unit interacts with another symmetry-related molecule, arranged in a face-to-face orientation in an alternate fashion (Figure 1). One molecule is rotated 60° with respect to its next neighbor, with the central rings superimposed and positioned at a distance of 3.56 Å. Short contacts within the two types of dimers involving the alkyl chains are indicative of CH-π interaction as has been previously observed in other triindole molecules, both in solution and in condensed state.^43−45^ Furthermore, as shown in Figure 1, multiple short C–H···S contacts at 2.88–3.24 Å can be found between the two units of each dimer, indicating the action of cooperative C–H···S hydrogen bonds in stabilizing the observed crystallographic packing.

**Figure 2:**
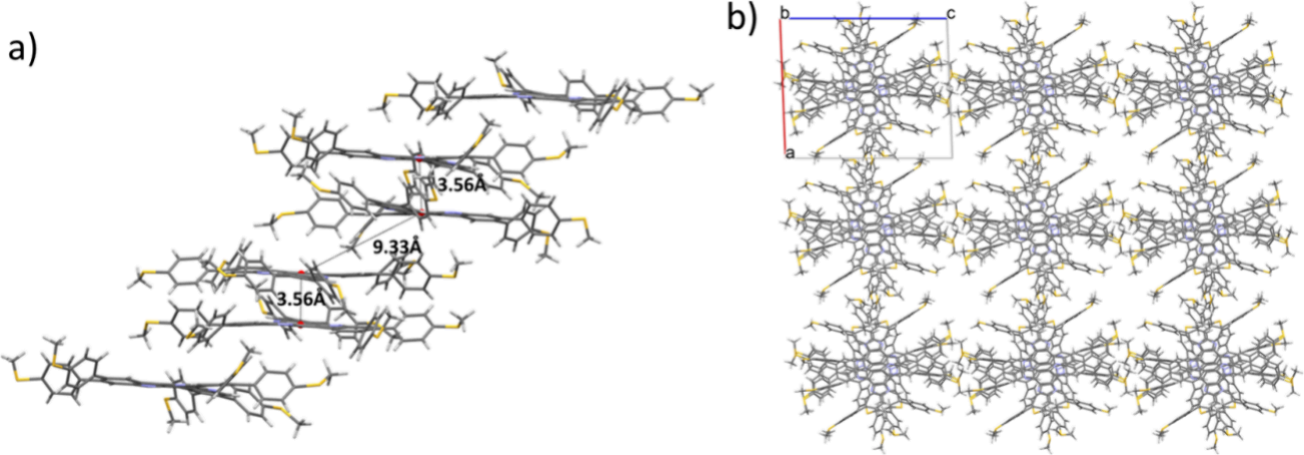
(a) Lateral view of the columns formed by the alternating dimers of molecules and (b) projection along the *b* axis of the columnar arrangement of compound **TRISMe**. The alkyl chains have been omitted for clarity.

A close analysis of the crystal packing shows the formation of a columnar arrangement in which the two types of dimers alternate forming tilted staggered stacks which extend along the *b* direction (Figure 2a). Crystal structure analysis evidence close contacts between the methylthio groups of molecules in different columns (Figure 2b). In the columns the distance between the centroids of next neighboring molecules, belonging to alternating dimers is large (9.33 Å), however numerous close contacts can be observed involving their external phenyl rings.

### Thermal Properties

The thermal properties of this compound have been studied by the combination of polarized optical microscopy (POM), differential scanning calorimetry (DSC), thermogravimetric analysis (TGA) and X-ray diffraction (XRD). As obtained polycrystalline **TRISMe** powder melts at 180 °C. Cooling from the isotropic liquid gives rise to a liquid crystalline mesophase that extends from 170 to 60 °C. At this temperature the compound does not crystallize but forms a glassy state that maintains the structural features of the mesophase presenting a glass transition that can be clearly observed by DSC (Figure S1). Upon heating the film show a cold crystallization at 140 °C before melting at 184 °C. These transition temperatures are observed in successive heating and cooling cycles. As could be determined by TGA (Figure S2), this compound shows a remarkable stability, with 2% weight loss temperature around 400 °C, well above the clearing temperature.

**Figure 3:**
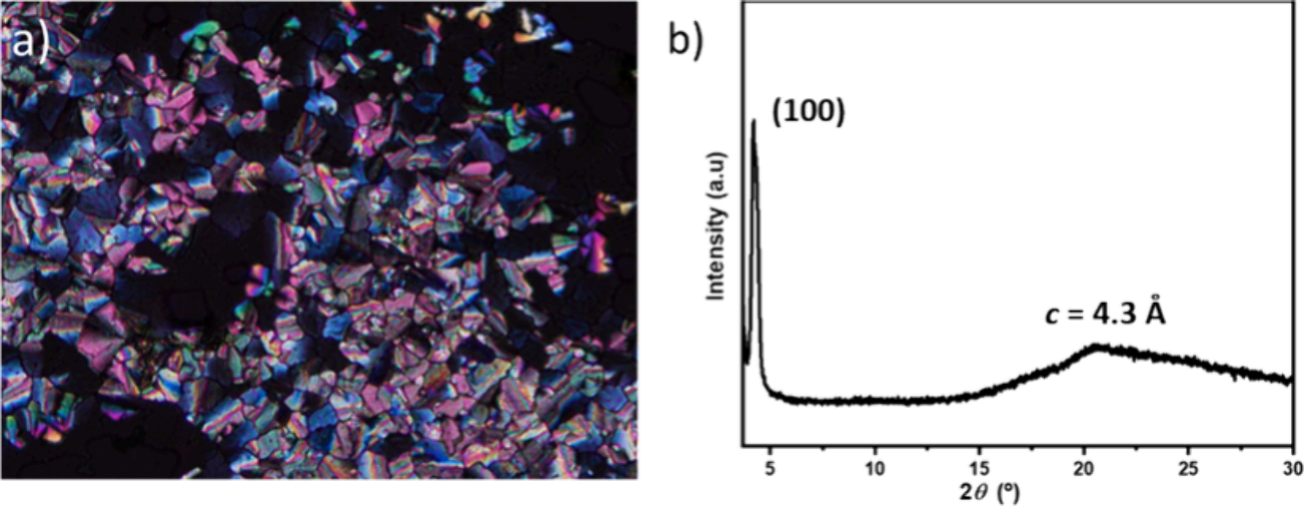
(a) Polarizing optical photomicrograph of **TRISMe** at 98 °C upon cooling from the isotropic liquid. (b) X-ray diffractogram of a film of **TRISMe** obtained after melting, annealing at 100 °C and rapid cooling to room temperature.

The monotropic mesophase was identified as hexagonal columnar (Colh) with a lattice constant *a* of 25 Å on the basis of its XRD data (Figure S3 and Table S1) and the typical pseudo focal conic fan-shaped texture observed by POM (Figure 3a). Interestingly, a rapid cooling of the mesophase to room temperature allows the freezing of this arrangement. In fact, the X-ray diffractogram of a film of **TRISMe** obtained after melting the compounds, annealing at the mesophase and rapid cooling to room temperature (Figure 3b) shows a sharp Bragg reflection at small angles which is indexed as 100 at 2θ = 4.21° and a broad diffuse scattering at medium angles indicative of the disordered alkyl chains. This diffractogram is in good agreement with a columnar hexagonal organization with a lattice constant *a =* 26.9 Å, very similar to that of the mesophase.

Comparison of the *a* and *c* unit cell parameters (19.53 and 24.32 Å respectively) of the crystal structure, with the *a* parameter of the columnar hexagonal mesophase (25 Å), suggests that upon heating the molecular fluctuations lead to an enhancement of the symmetry of the system by reducing the lateral displacements of the two dimers. The more expanded 2D unit cell of the hexagonal mesophase also suggests a decrease in the tilt of the molecules with respect to the columnar axis. Presumably, in the mesophase the polarizable sulfur atoms provide dispersive attractive interactions, that together with the flexibility and geometrical features imposed by the C–S bonds provide the necessary steric perturbation between columns which contribute to stabilize the discotic mesophases. Note that although terminal thiomethyl groups attached to rod-shaped molecules have been proposed to be “good smectogenic groups”,^46^ to our knowledge this is the first report of a columnar mesophases promoted by peripheral thiomethyl groups in disk-shaped molecules.

### Charge Transport Measurements and Film Morphology

The promising hole transport properties previously reported for triindole-based discotic liquid crystals,^36−39^ together with the lack of long peripheral alkyl chains in this new triindole mesogen, renders **TRISMe** attractive in the search of discotic liquid crystals with low alignment dependence. With this in mind, we have studied the charge transport properties of **TRISMe** using two different methods, each with opposite alignment requirements: space charge limited current (SCLC) measurements^47^ in a diode-type structure, (requiring a conducting channel perpendicular to the electrodes) and field effect mobility in organic field-effect transistor (OFET) devices,^48^ (requiring a conducting channel parallel to the dielectric substrate). These devices could be easily prepared by melting and solution processing, respectively.

SCLC measurements were performed on a diode structure consisting on films of **TRISMe** sandwiched between an Au electrode and an ITO electrode. **TRISMe** was introduced by capillarity from its isotropic melt, slowly cooled to reach the mesophase and subsequently rapidly cooled to room temperature to maintain the characteristics of the mesophase. The use of gold as positive electrode ensures the efficient hole injection, as Au work function is −5.1 eV which matches well with the HOMO value of the triindole material (−5.08 eV as estimated by cyclic voltammetry, see Figure S4) which is necessary in order to avoid major underestimations of mobility values. ITO was employed as a counter electrode because of its work function of ∼ −4.6 eV, much lower than the estimated LUMO energy of **TRISMe (**–2.01 eV, as estimated from the difference between the HOMO level and the optical gap, see Section 6 in the Supporting Information) and because its transparency allows checking the orientation and texture of the liquid crystal by POM.

When measuring currents as a function of the applied voltage, at low fields an ohmic regime is usually found, with the current depending linearly on the applied voltage. At higher fields a space-charge field may be present and, neglecting the effect of traps, the current follows the Mott–Gurney equation^49^ (1):
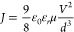
1where *J* is the measured current density, μ is the charge mobility, ε_0_ is the free space permittivity, ε_r_ is the relative dielectric constant of the material, *V* is the applied voltage and *d* is the thickness of the sample. From such equation, since the relative dielectric constant ε_r_ and the sample thickness *d* can be easily measured, it is possible to obtain the charge carrier mobility μ.

**Figure 4:**
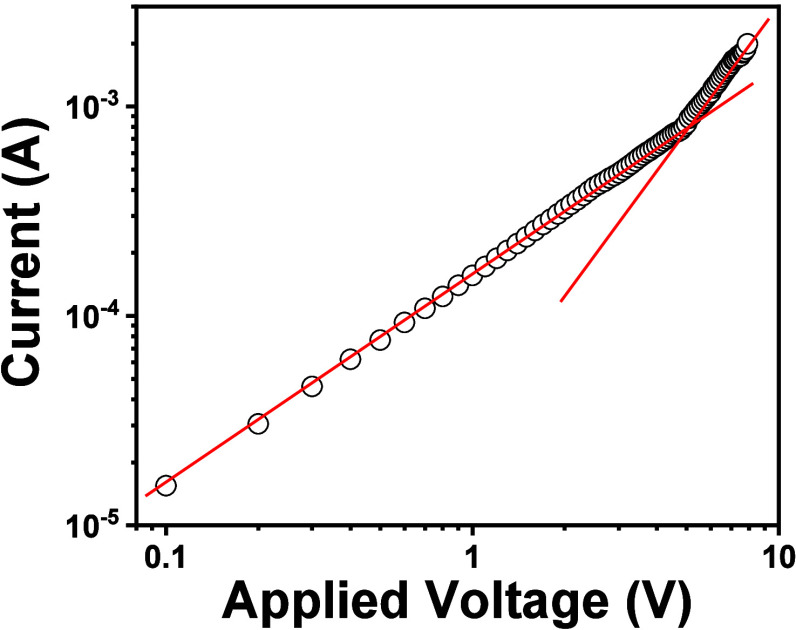
Typical current/voltage characteristic curve (symbols) obtained from a 0.6 mm^2^, 8 μm thick sample of **TRISMe**. The two lines are not fittings but show slopes 1 and 2 in the double logarithmic graph.

Prior to the measurement, cells were subjected to a thermal annealing process at the temperature of the mesophase and subsequently cooled down to room temperature in order to maintain the characteristics of the mesophase (Figure S6). It was possible to observe the behavior predicted by eq 1 and extract a value of hole mobility in about one-third of the different measurement areas of each sample. This may be attributed to difficulties in obtaining good injection or to the small dimension of different orientational domains, with the consequent formation of grain boundaries acting as charge traps. Figure 4 shows one of the typical characteristic curves that allowed the measurement of mobility. The average mobility over the different areas of different devices was 3 × 10^–1^ cm^2^V^–1^ s^–1^. Note that closely related hexaaryltriindole based liquid crystals functionalized with six nonyl chains, have led to mobility values several orders of magnitude lower, highlighting the beneficial effect of the thiomethyl groups.^36^

**Figure 5:**
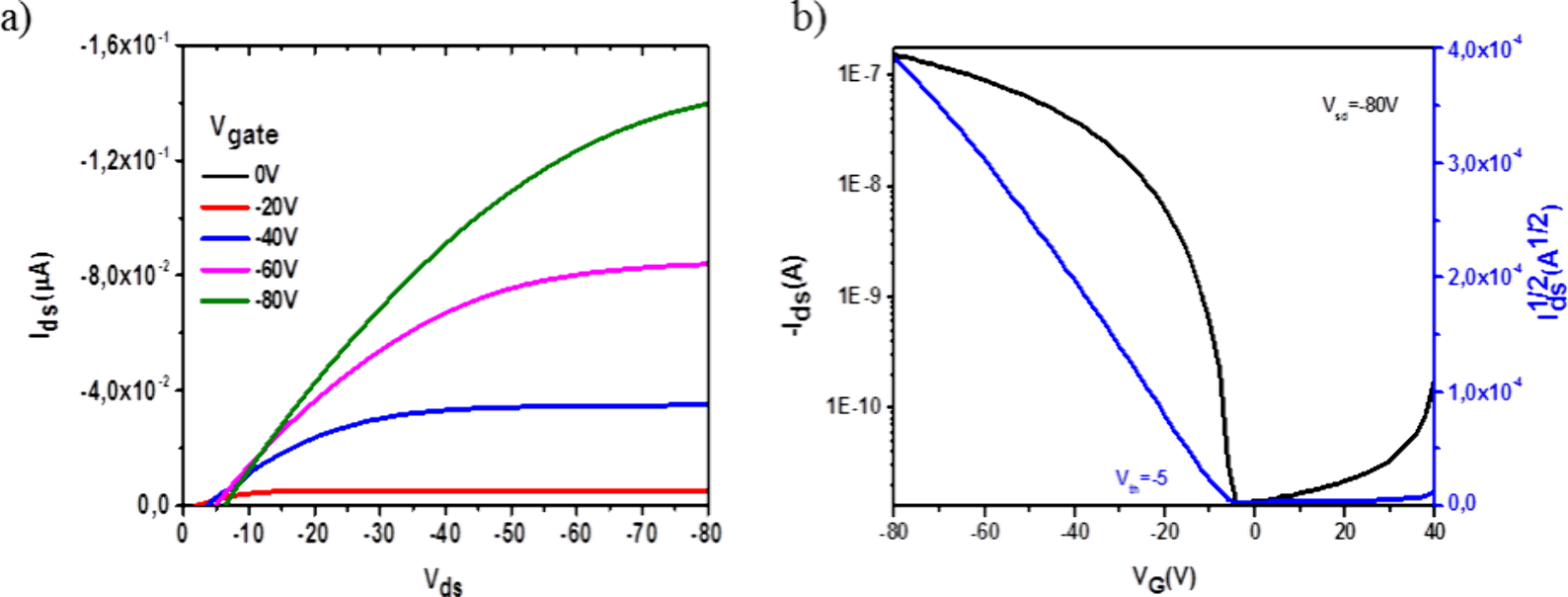
(a) Output curves of **TRISMe** measured at gate voltages from 0 to −80 V in intervals of 20 V. (b) Transfer curves of **TRISMe** measured at a source-drain voltage of −80 V.

Furthermore, we have measured the mobility of this semiconducting mesogen in a field effect transistor device in crystalline films. Thin-film transistors were fabricated in a bottom gate, top contact configuration by drop-casting a 10 mL of a chloroform solution of **TRISMe** (5 mg/mL) on HMDS-functionalized Si/SiO_2_ substrate, followed by evaporation of gold source/drain electrodes through a shadow mask. Charge transport evaluation was carried out via analysis of the OFET current–voltage response in the saturation regime, following eq 2.^50^
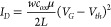
2where *W* and *L* are the channel width and length, respectively, *C*_*ox*_ is the dimensional dielectric capacitance of gate insulator, μ is the hole mobility and *V*_*th*_ is the threshold voltage. For the calculation of the relevant OFET parameters, we used transfer plots of *I*_*DS*_ vs. *V*_*G*_. Figure 5a reports the output curves of **TRISMe** measured at gate voltages from 0 to −80 V in intervals of −20 V. Figure 5b shows the transfer characteristics of **TRISMe** measured at a fixed source-drain voltage of −80 V. A mobility of 3.01 × 10^–4^ cm^2^V^–1^s^–1^ with a threshold voltage close to −5 V were measured with this technique.

**Figure 6:**
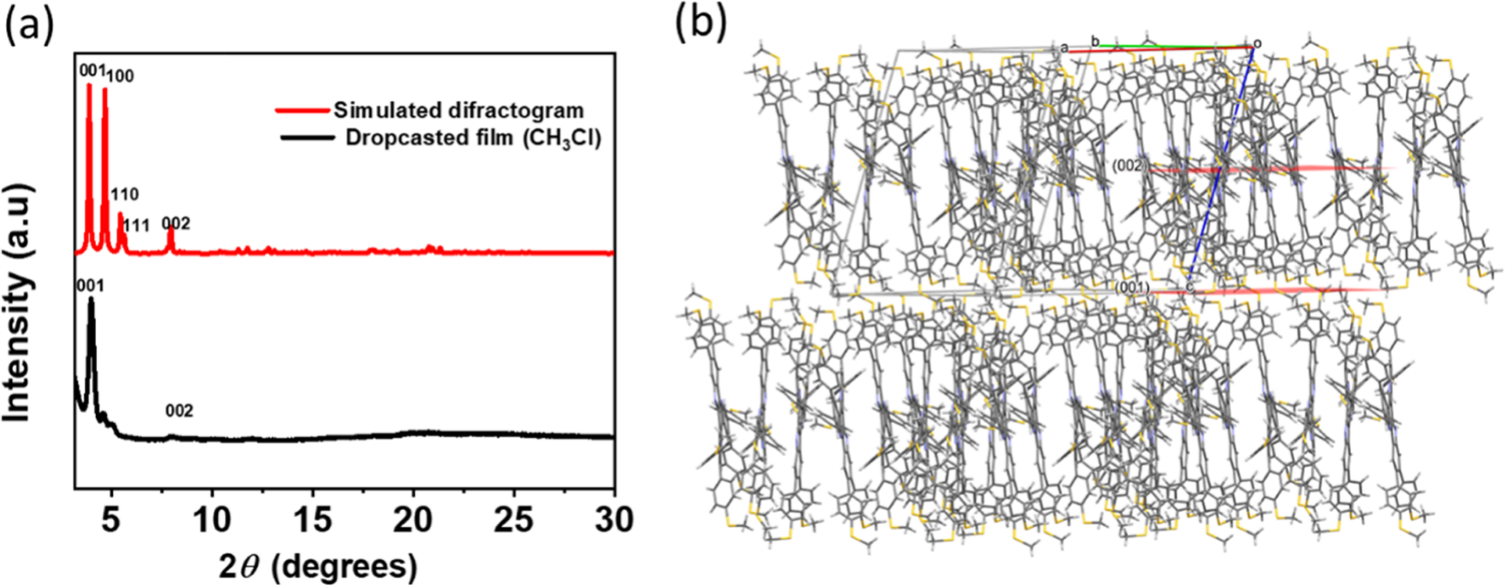
(a) Comparison of the powder X-ray diffractograms of the films of **TRISMe** prepared by drop-casting and the diffractogram simulated from the single crystal X-ray data. (b) columnar packing showing the (001) and (002) planes in red.

In order to get information on the arrangement of the molecules in the films and correlate it to charge transport in the OFET, we performed X-ray diffraction on films prepared by drop-casting of a chloroform solution of **TRISMe**, mimicking the processing conditions used in the fabrication of the devices. Sample diffractogram is dominated by an intense broad peak at 2θ = 4.02°, d = 21.95 Å which coincides within the experimental error with the one indexed as (001) in the powder diffractogram simulated from the single-crystal data of **TRISMe** (2θ = 3.93°, d = 22.45 Å), although the broadening in the peak indicates a reduction of the crystallinity (Figure 6). Some minor peaks, are also observed probably corresponding to the presence of other the minor polymorphs. The fact that only one peak can be observed in the X-ray diffractogram of films suggests that molecules present a preferred orientation with their (001) faces aligned parallel to the surface of the XRD sample holder. Note that a higher order reflection at 2θ = 8.04°, d = 10.91 Å can be also observed).

Although such alignment would provide a charge transport plane parallel to the surface, as needed to have an organic field effect transistor (OFET) behavior, the lower mobility found is probably due to a less favorable supramolecular arrangement in spite of the higher crystallinity. Crystallization leads to and enhancement of order, but also to an unfavorable alternation of the dimers in the columns. In fact, annealing the device for 5 min at 150 °C significantly enhances the crystallinity of the film (Figure S7), but the OFET mobility values decreases 1 order of magnitude (Figure S8).

## Conclusion

In conclusion, our research introduces **TRISMe**, a novel triindole-based semiconducting discotic liquid crystal functionalized with six peripheral *p*-methylthiophenyl groups which successfully impede tight molecular packing while promoting a stabilized columnar arrangement within this disk-shaped molecule. In fact, this compound exhibits a columnar mesophase over a wide temperature range despite being endowed with only three flexible alkyl chains attached to the nitrogens. The methylthio groups play also a pivotal role in the growth and packing of crystals of **TRISMe**, resulting in the formation of dimers stabilized by C–H···S interactions which further alternate to generate slipped columns. The semiconducting properties of **TRISMe** have been studied by estimating hole mobility of this mesogen by means of two different methods: through space charge limited current measurements in a diode-type device with the semiconductor in a supercooled columnar mesophase showing a mobility value of 3 × 10^–1^ cm^2^ V^–1^ s^–1^ or via field effect mobility measurements in a thin film transistor (measuring 3 × 10^–4^ cm^2^ V^–1^ s^–1^ ) wherein the drop-casted semiconductor organizes in crystalline films with the columnar axis aligned parallel to the substrate surface. The higher mobility observed in the SCLC device suggests that the molecular fluctuations in the mesophase enhance the symmetry of the system and reduces the alternation of the dimers in the columns, thereby facilitating charge transport. However, the observation of field effect behavior in this compound, even when processed via straightforward drop-casting, implies a significant tolerance to diverse columnar alignments within the crystalline film. We attribute this high tolerance, uncommon in materials with highly uniaxial columnar superstructures typical of discotic mesogens, to the low isolating versus conducting ratio within this mesogen. The result of this study not only demonstrates the practical potential of **TRISMe** for its easy integration into solution-processable electronic devices, but also highlights the thiomethyl moieties as potent motifs for the development of easily processable columnar superstructures endowed with semiconducting properties.

## Supporting Information Available

Additional experimental details on the characterization of **TRISMe** (NMR spectra, details on mesophase characterization, cyclic voltammetry measurements, UV–vis measurements, POM of the diode-type device, comparison of simulated and experimental X-ray diffractograms, and transfer curves after thermal annealing (PDF)
CIF file of compound **TRISMe** (CIF)

## Supplementary Material

el4c00693_si_001.pdf

el4c00693_si_002.cif
