# Supplementary material for: Columnar Mesomorphism in a Methylthio-Decorated Triindole for Enhanced Charge Transport
Source: ACS Appl Electron Mater. 2024 Jun 11;6(6):4709–17. doi: 10.1021/acsaelm.4c00693 (PMC11210202; doi:10.1021/acsaelm.4c00693)
Supplement: Supplementary file 1 — el4c00693_si_001.pdf [file el4c00693_si_001.pdf]

Supporting Information

Columnar Mesomorphism in a Methylthio-decorated Triindole for Enhanced Charge Transport

Constanza Ruiz[1,2], Raúl Martín[1,3], Angela Benito[1], Enrique Gutierrez[1], M. Ángeles Monge[1], Antonio Facchetti,[2] Roberto Termine[4], Attilio Golemme[4], Berta Gómez-Lor[1,]*

1 Instituto de Ciencia de Materiales de Madrid, CSIC, Cantoblanco, 28049, Madrid, Spain

2 School of Materials Science and Engineering, Georgia Institute of Technology, Atlanta, Georgia 30332, USA

3 Faculty of Chemical and Technologies Sciences, University of Castilla-La Mancha.13071 Ciudad Real, Spain.

4 CNR Nanotec UOS Rende, Dipartimento di Fisica, Università della Calabria, Rende, 87036, Italy

Corresponding Author

* E-mail: bgl@icmm.csic.es, Phone: (+34) 91-3349031, Fax: (+34) 91-3720623

Content

1. Copy of ^{1}H NMR and ^{13}C NMR spectra of compound TRISMe 3
1.1 Copy of 1H NMR spectrum (200 MHz, CDCl3) 3
1.2 Copy of 13C NMR spectrum (50 MHz, CDCl3) 4
2. Liquid crystalline techniques and experimental details 4
3. Differential Scanning Calorimetry traces of compound TRISMe 6
4. Thermogravimetric analysis of compound TRISMe 6
5. X-Ray diffraction details of the mesophase 7
6. Cyclic voltammetry 7
7-. Absorption spectra 9
8. Polarizing optical photomicrograph of TRISMe in the diode-type device 9
9. Comparison of simulated powder X-ray diffraction patterns and experimental difractogram of TRISMe after thermal annealing. 10
10. Transfer curves of TRISMe after annealing at 150ºC 10
11. References. 11

1. Copy of ^{1}H NMR and ^{13}C NMR spectra of compound TRISMe

1.1 Copy of ^{1}H NMR spectrum (300 MHz, $C_2D_2Cl_4$)

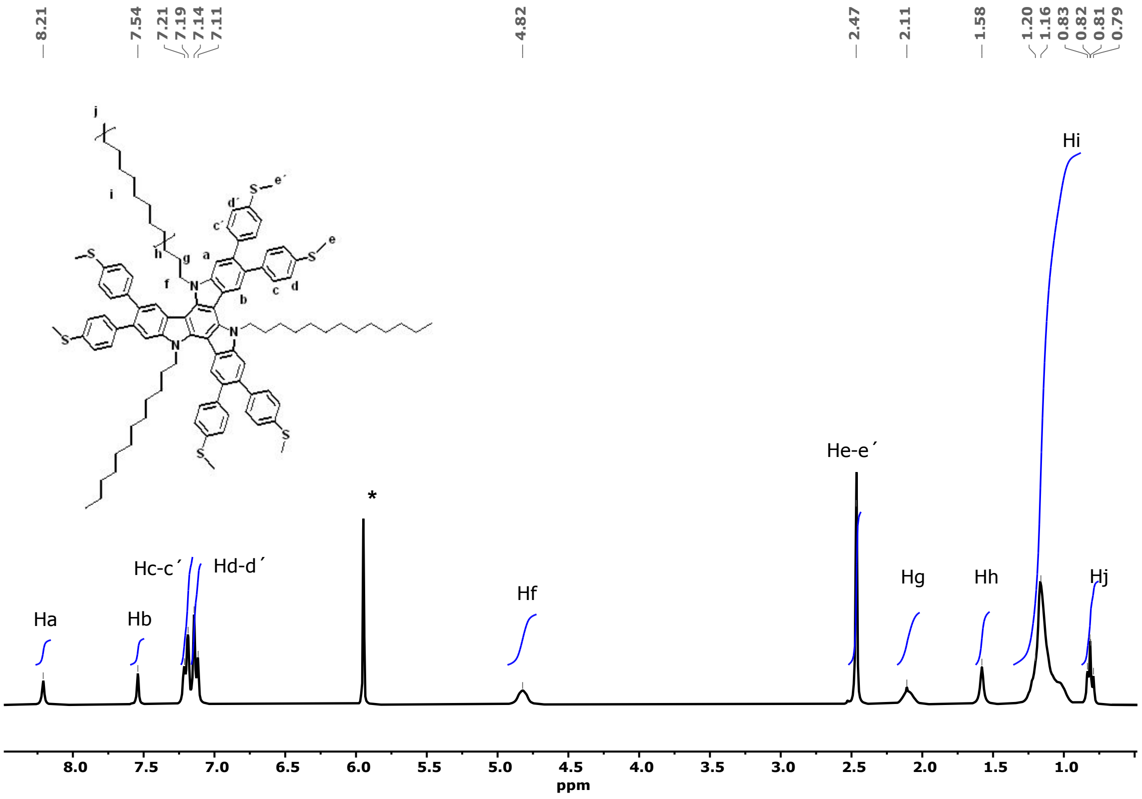

^{1}H NMR (300 MHz, $C_2D_2Cl_4$, 25 °C, ppm) δ 8.21 (s, 3H, H_a), 7.54 (s, 3H, H_b), 7.21-7.19 (AA´BB´, 12H, $H_{c-c'}$), 7.14-7.11 (AA´BB´, 12H, $H_{d-d'}$), 4.82 (t, 6H, H_f), 2.47 (s, 18H, $H_{e-e'}$), 2.11 (m, 6H, H_g), 1.58 (m, 6H, H_h), 1.20-1.16 (m, 48H, H_i), 0.83-0.79 (m, 9H, H_j).

1.2 Copy of ^{13}C NMR spectrum (75 MHz, $CDCl_3$)

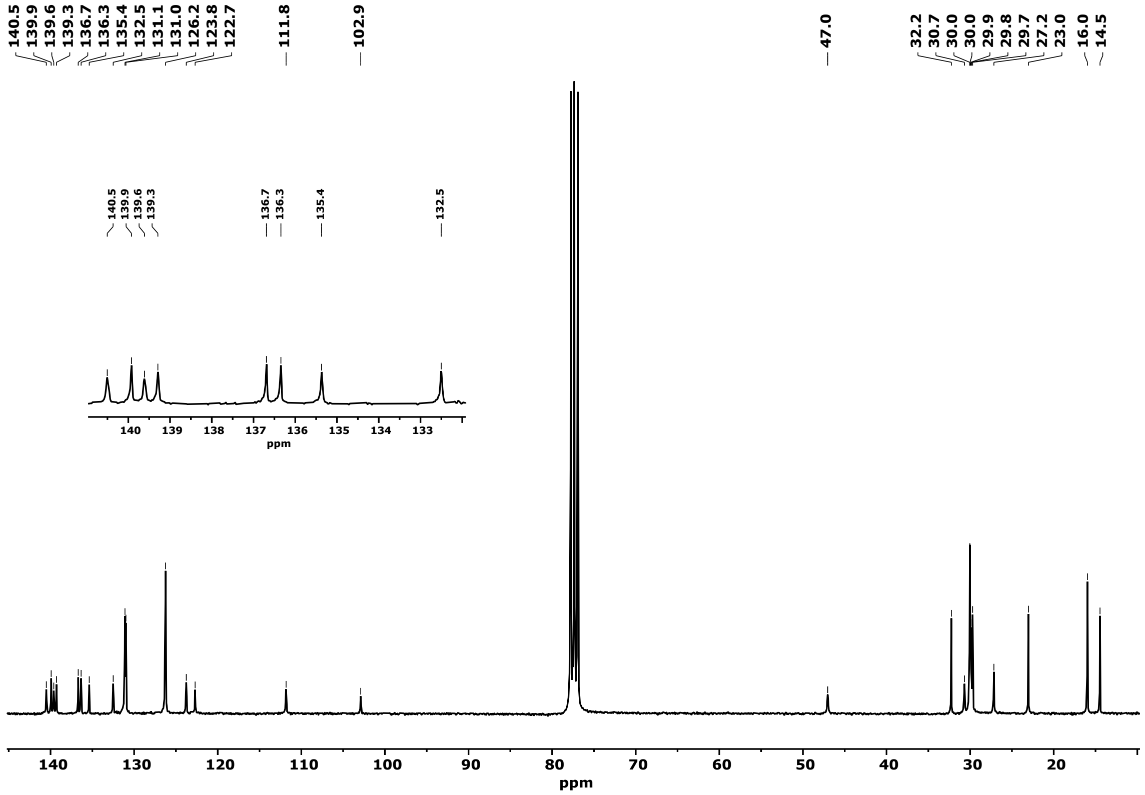

^{13}C NMR (75 MHz, $CDCl_3$, 25 °C, ppm) δ 140.5 (C), 139.9 (C), 139.6 (C), 139.3 (C), 136.7 (C), 136.3 (C), 135.4 (C), 132.5 (C), 131.1 (CH), 131.0 (CH) 126.2 (CH), 123.8 (CH), 122.7 (C), 111.8 (CH), 102.9 (C), 47.0 (N-CH_2), 32.2 (-CH_2), 30.7 (CH_2), 30.0 (-CH_2), 30.0 (-CH_2), 29.9 (-CH_2), 29.8 (-CH_2), 29.7 (-CH_2), 27.2 (-CH_2), 23.0 (-CH_2), 16.0 (S-CH_3), 14.5 (-CH_3).

2. Liquid crystalline techniques and experimental details

The optical textures of the mesophases were studied with a Nikon polarizing microscope Eclipse LV 100N POL equipped with a Linkam hot-stage and Linkam LINKSYS32 central processor and microphotographs were taken with a Nikon DP12 digital camera. The transition temperatures and enthalpies were measured by differential scanning calorimetry with a DISCOVERY DSC Q100 calorimeter operated at a scanning rate of 10 °C min^{-1} on both heating and cooling and under atmosphere of N_2. Thermogravimetric analysis (TGA) was performed to determine the thermal stability of the compound and was carried out on a TA TGA Q500 analyzer under a N_2 atmosphere. The sample was

heated at 10 ºC min^{-1} from room temperature to 850 ºC. The XRD patterns at the mesophase were obtained with a pinhole camera (Anton-Paar) operating with a point-focused Ni-filtered Cu-Kα beam. The sample was held in Lindemann glass capillaries (1 mm diameter) and heated, when necessary, with a variable-temperature oven. The capillary axis is perpendicular to the X-ray beam and the pattern is collected on flat photographic film perpendicular to the X-ray beam. Spacing was obtained via Bragg's law. The XRD pattern of the cooled mesophase was obtained with a Bruker D8 Advance diffractometer with a Sol-X energy dispersive detector, working at 40 kV and 30 mA and employing CuK_{α} (λ = 1.5418 Å) filtered radiation. The diffractograms were registered with a step size of 0.02 º and exposure time of 0.5 s per step and a 2θ range of 2.5-30 º.

3. Differential Scanning Calorimetry traces of compound TRISMe

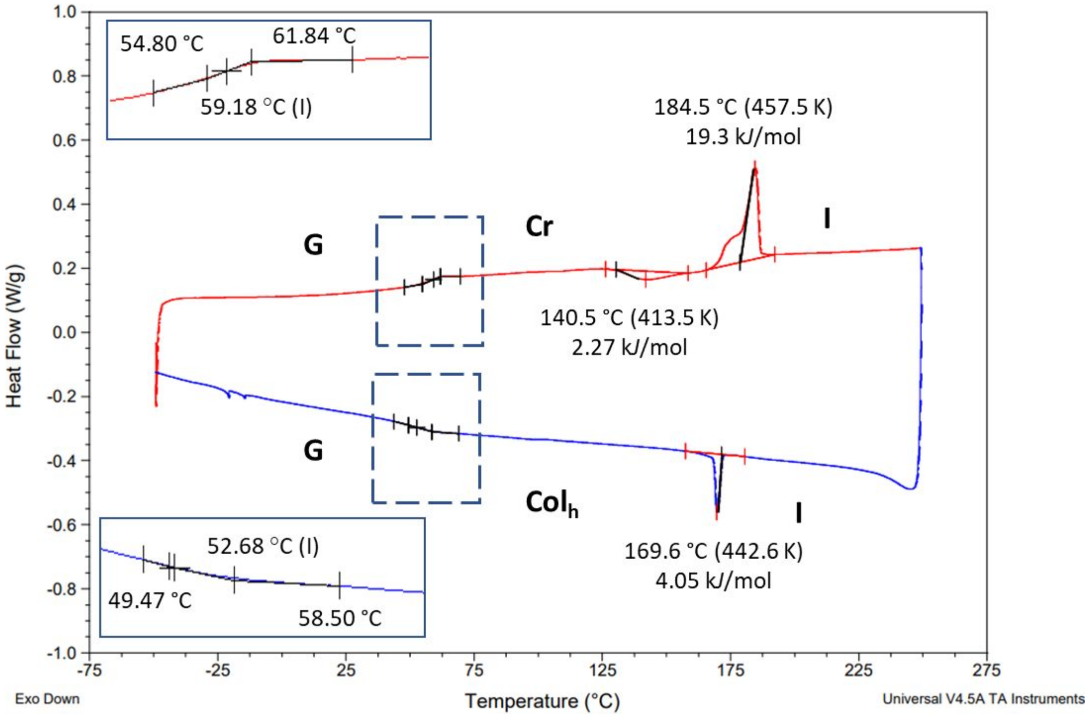

Figure S1. DSC traces of the compound **TRISMe** corresponding to the second scan (10 ºC min^{-1}, Exo down). G:glassy, I: isotropic liquid, Col_h: hexagonal columnar phase, Cr: crystal.

4. Thermogravimetric analysis of compound TRISMe

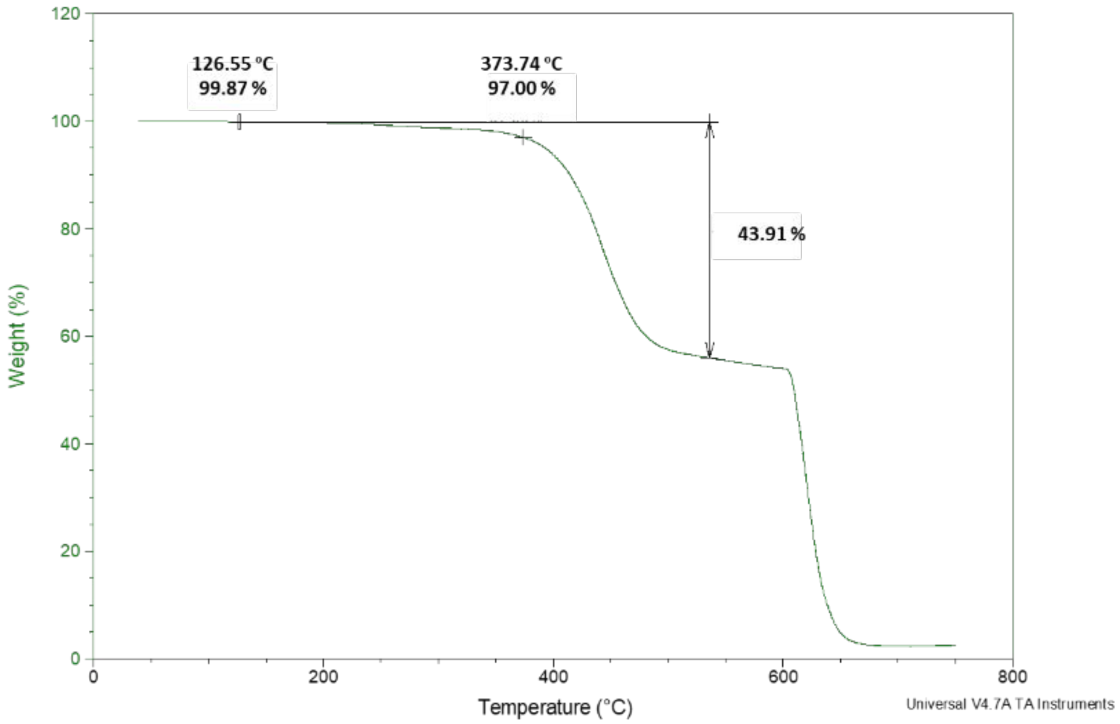

Figure S2. Thermogravimetric analyses (TGA) of compound **TRISMe**

5. X-Ray diffraction details of the mesophase

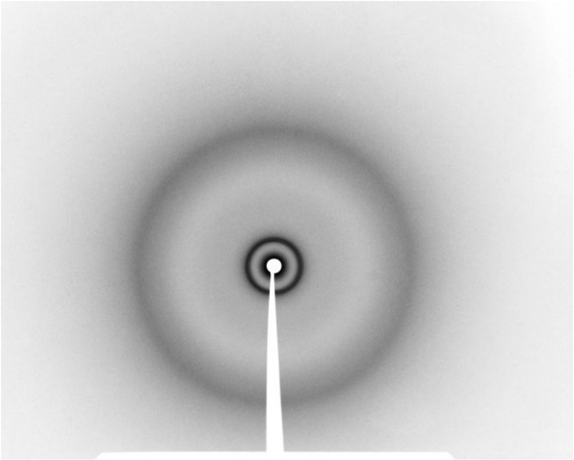

Figure S3. X-ray pattern of the hexagonal columnar mesophase of **TRISMe** recorded at 80ºC

Table S1. X-ray results for the mesophase of **TRISMe**.

Temperature (ºC)	Mesophase	*h k l*	d_{obs} (Å)	d_{calc} (Å)	Lattice constants (Å)
80ºC	Col_h	1 0 0	21.65	21.65	*a* = 25.0
			4.67 [a]		
ta	Col_h	1 0 0	21.53	21.53	*a* = 24.9
			4.64 [a]		

[a] Diffuse, broad maximum.

6. Cyclic voltammetry

Cyclic voltammetry (CV) experiments were performed on a Bioanalytical Systems Inc. (BASI) Epsilon electrochemical workstation in a three-electrode cell at room temperature under nitrogen atmosphere. Electrochemical measurements were carried out in CH_2Cl_2 solution (c = 1 x 10^{-3} M) containing 0.1 M tetra-n-butylammonium hexafluorophosphate ($TBAPF_6$) of supporting electrolyte at a scan rate 100 mV/s. A three electrode setup was used including a platinum working electrode, Ag/AgCl (3 M NaCl) reference electrode,

and a platinum wire auxiliary electrode. Ferrocene was used as an internal standard, an all potentials were referenced to the ferrocene/ferrocenium redox couple.

The HOMO energy values of **TRISMe** was estimated as -5.08 eV from the first oxidation potential with respect ferrocene/ferrocenium redox couple[1] and considering a value of -4.8 eV for Fc with respect to zero vacuum level. This value is obtained from the calculated value of - 4.6 eV for the standard electrode potential (E) using a normal hydrogen electrode (NHE) on the zero vacuum level and the value of 0.2 V for Fc vs. NHE.[2,3]

The LUMO energy values was estimated from the difference between the HOMO level and the optical gap (3.07 eV), which render a LUMO level of -2.01 eV.

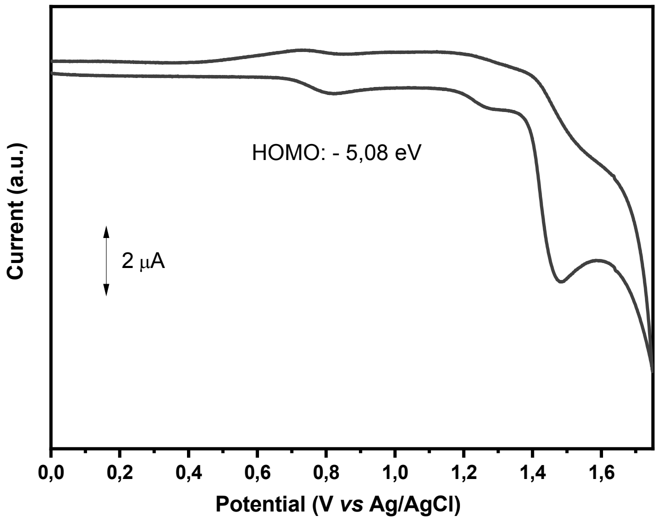

Figure S4. Measured cyclic voltammogram of compound **TRISMe** (c = 1×10^{-3} M) recorded at a scan rate of 100 mV.s^{-1} in CH_2Cl_2/0.1 M Bu_4NPF_6 as electrolyte.

7-. Absorption spectra

UV-vis studies were carried out on a PerkinElmer Lamba XLS+ spectrometer.

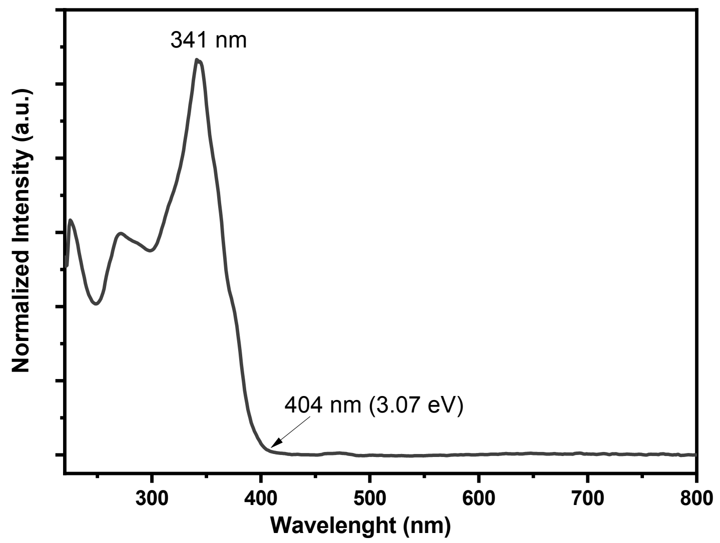

Figure S5. Experimental UV-vis absorption spectra of **TRISMe** in a 10^{-5} M CH_2Cl_2 solution.

8. Polarizing optical photomicrograph of TRISMe in the diode-type device

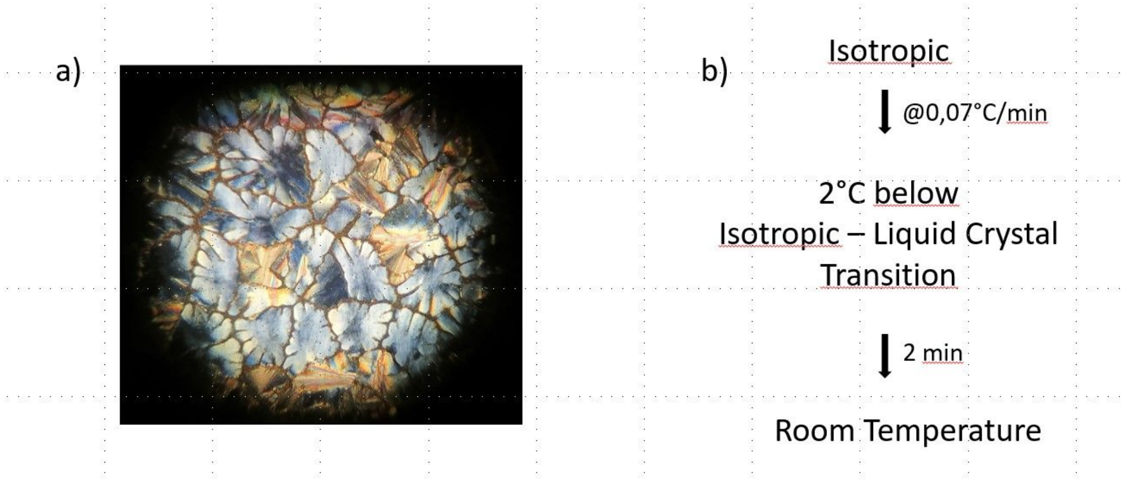

Figure S6. a) Polarizing optical photomicrograph of **TRISMe** at room temperature upon cooling from the isotropic liquid, following the thermal treatment shown in b).

As it can be seen, the annealed samples used for SCLC measurements were not uniformly aligned. However, as it was possible to observe by rotating the samples between crossed polarizers, several areas (orientational domains) were of a gray or almost black shade changing little with rotation, while other areas were much brighter and showed a much higher brightness variation with rotation. This indicates that in each measurement areas there were several

homeotropic or almost homeotropic domains. As the anisotropy of charge mobility in columnar mesophases is estimated to be around 3-4 orders of magnitude, the contribution of non-homeotropic domains to the measured current is minor and one can assume that most of the SCLC signal derives from intracolumnar mobility.

9. Comparison of simulated powder X-ray diffraction patterns and experimental difractogram of TRISMe after thermal annealing.

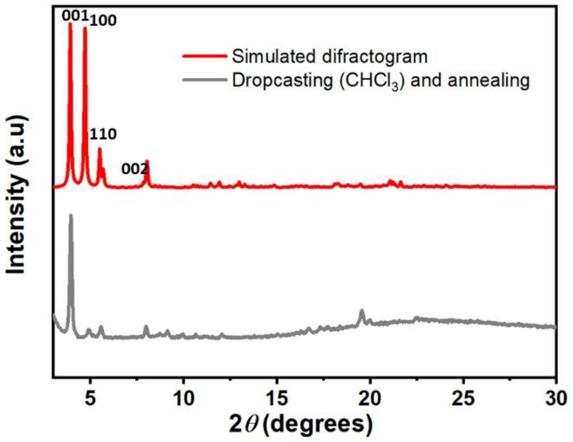

Miller index	Simulated		Experimental	
	2θ (°)	d (Å)	2θ (°)	d (Å)
001	3.94	22.40	3.97	22.23
100	4.72	18.70	4.86	18.16
110	5.49	16.05	5.57	15.84
002	7.88	11.20	7.97	11.07

Figure S7. Comparison of the experimental powder X-ray diffractograms of the films of **TRISMe** prepared by drop-casting and annealed 5 min at 150ºC (gray) and the diffractogram simulated from the single crystal X-ray data (red).

10. Transfer curves of TRISMe after annealing at 150ºC

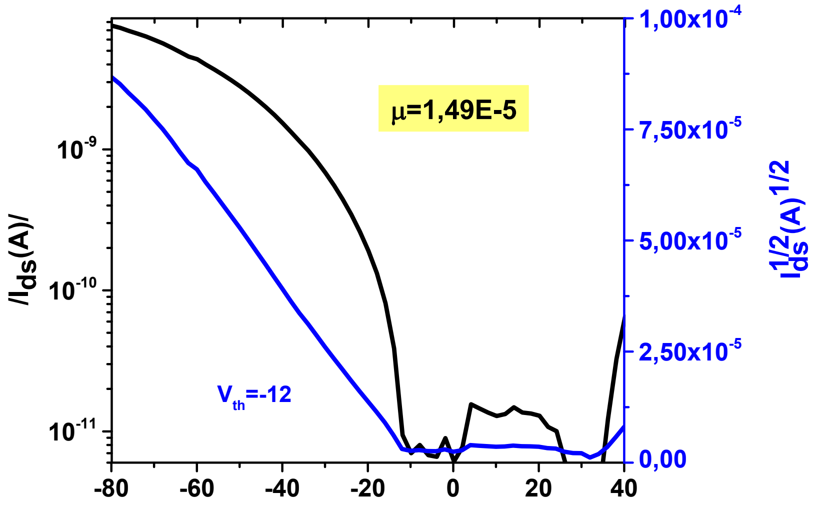

Figure S8. Transfer curves of **TRISMe** after annealing the device for 5 min. at 150 ºC measured at a source-drain voltage of -80V

11. References.

1. Gritzner, G.; Kuta, J. Recommendations on Reporting Electrode-Potentials in Nonaqueous Solvents. *Pure Appl. Chem.* **1984**, *56*, 461-466.

2.Bard, A. J.; Faulkner, L. R.; Wiley, Ed. New York, 1980.

3. Trassati, S. The Absolute Electrode Potential: An Explanatory Note. *Pure Appl. Chem.* **1986**, *58*, 955-966.
